# Does Chronic Intestinal Inflammation Promote Atrial Fibrillation: A Mendelian Randomization Study With Populations of European Ancestry

**DOI:** 10.3389/fcvm.2021.641291

**Published:** 2021-05-10

**Authors:** LaiTe Chen, ChenYang Jiang

**Affiliations:** Department of Cardiology of Sir Run Run Shaw Hospital, School of Medicine, Zhejiang University, Hangzhou, China

**Keywords:** atrial fibrillation, inflammatory bowel disease, Mendelian randomization, single nucleotide polymorphisms, risk factor

## Abstract

**Background:** Inflammatory bowel disease (IBD), comprising ulcerative colitis (UC), and Crohn's disease (CD), has been reported to be associated with an increased risk of atrial fibrillation (AF). However, the causal role of the chronic intestinal inflammation (CII) in the development of AF remains controversial. We use Mendelian randomization (MR) analysis to explore the causal inference of CII on AF.

**Methods:** A two-sample MR analysis was performed to estimate the potential causal effect of CII on AF. Statistical summaries for the associations between single nucleotide polymorphisms (SNPs) and phenotypes of CII were obtained from genome-wide association studies (GWAS) with cohorts of CD (*n* = 51,874), UC (*n* = 47,745), and IBD (*n* = 65,642) of European descent. The GWAS of 1,030,836 people of European ancestry, including 60,620 AF cases and 970,216 controls was collected to identify genetic variants underlying AF. The causal inference was estimated using the multiplicative random effects inverse-variance weighted method (IVW). The methods of MR-Egger, simple median, and weighted median were also employed to avoid the bias of pleiotropy effects.

**Results:** Using three sets of SNPs (75 SNPs of CD, 60 SNPs of UC, and 95 SNPs of IBD), multiplicative random-effect IVW model estimated a universal null effect of CII on AF (CD: OR = 1.0059, 95% CI: 0.9900, 1.0220, *p* = 0.47; UC: OR = 1.0087, 95% CI: 0.9896, 1.0281, *p* = 0.38; IBD: OR = 1.0080, 95% CI: 0.9908, 1.0255, *p* = 0.37). Similar results were observed using the MR-Egger, simple median, weighted median methods.

**Conclusion:** As opposing to the traditional observational studies, our two-sample MR analysis did not find enough evidence to support a causal role of either CD or UC in the development of AF.

## Introduction

It has long been established that inflammation plays a vital role in the pathological progress of atrial fibrillation (AF). The activated NLRP3 receptor promotes autophagy and mitophagy, which lead to impaired mitochondrial function and reactive oxygen species, contributing to premature atrial beats that may initiate episodes of AF (1). Interleukin-6 and interleukin-8, as pro-inflammatory cytokines, may disrupt the structure of the atrial myocardium, resulting in slow conduction, which in turn maintains the burst of AF (2). Given the implication of inflammation in the development of AF, the assumption of AF susceptibility in inflammatory disease has been made and verified. Compared with age- and sex-matched general patients, systemic lupus erythematosus was associated with a doubled rate of hospitalization for AF (3). A systematic review and meta-analysis have revealed an increased risk ratio of 1.29 (95% CI, 1.05–1.59) in the development of AF in patients with rheumatoid arthritis, as compared to the control group (4).

As a state of chronic intestinal inflammation (CII), inflammatory bowel disease (IBD), comprised of ulcerative colitis (UC) and Crohn's disease (CD), was also reported to be associated with AF incidence in several studies. A retrospective study showed that AF has an overall higher prevalence across all age groups in the IBD population (5). During the follow-up of a population-based cohort study, patients with IBD were identified at a 36% (95% CI, 20–54%) higher risk of AF than controls (6). On the contrary, using the nationwide inpatient database in the United States, patients with IBD were found less likely to be hospitalized for arrhythmias than the non-IBD (9.7 vs. 14.2%, *p* < 0.001) (7). The potential biases of confounding factors and reverse causation may contribute to such controversy (8), leaving the specific relationship between IBD and AF yet to be determined.

Given that the exposure-associated genetic variants are randomly distributed and not subject to reverse causation, Mendelian randomization (MR) serves as a valuable approach to assess the causal inference of an observed association between a modifiable exposure and a clinically relevant outcome (9). Therefore, we applied MR analysis to explore the potential causal association between CII and AF in this study.

## Methods and Materials

### Study Design and Data Sources

Valid estimates could be explored from two-sample MR analyses when three key assumptions are met: (1) the instrumental variants (SNPs) are associated with the exposure (CD, UC, and IBD); (2) the instruments may impact the outcome (AF) only via their effects on exposure; and (3) the instruments are independent of any confounders for the association between exposure and outcome.

Candidate genetic instruments for CII were selected from genome-wide association studies (GWAS). In terms of the radiologic, endoscopic, and histopathologic evaluation, cases that fit the clinical criteria for CII phenotypes (CD, UC) and matched controls of European ancestry were recruited. To prevent the pleiotropic bias from trans-ancestry cases (10), all individuals in this study were of European ancestry. Following quality-control, the cohorts of CD (*n* = 51,874), UC (*n* = 47,745), and IBD (*n* = 65,642) of European descent were used to carry out the GWAS (11) (Table 1). The diagnosis of CD and UC was made at the age of 28.39 ± 14.16 and 34.10 ± 15.78 years old. Males accounted for 45.1% of CD and 52.1% of UC patients. Nearly half of CD cases (46.8%) had ileocolonic lesions while 48.9% of UC cases had extensive lesions. Over half of CD patients (52.8%) received abdominal surgery whereas only 18.5% of UC patients took colectomy. To derive the causal effect of CII on AF, three sets of genetic instruments (CD, UC, and IBD) were extracted from combined summary statistics of GWAS.

**Table 1:** Description of contributing studies.

**Contribution**	**Traits**	**Sample size**	**Number of SNPs**	**Author**	**Population**
Exposure	Crohn's disease	51,874	124,888	Rinse KW	European
	Ulcerative colitis	47,745	156,116	Rinse KW	European
	Inflammatory bowel disease	65,642	157,116	Rinse KW	European
Outcome	Atrial fibrillation	1,030,836	33,519,037	Nielsen JB	European

### Selection of Genetic Instrumental Variants

With 17,897 CD cases, 13,768 UC cases, and 33,977 controls, this European GWAS reported 124,888, 156,116, and 157,116 single nucleotide polymorphisms (SNPs) for phenotypes of CD, UC, and IBD, respectively (11) (Table 1). For the first key assumption of MR analysis, since the selected SNPs were identified through the GWAS, such instruments are likely to be associated with three sets of exposures (CD, UC, and IBD) in the source population. The protocol for the selection of genetic instrumental variants contained three steps. During the preliminary selection, parameters used to identify candidate instrumental variants included a *p*-value of 5 × 10^−8^ for genome-wide significance, the minor allele frequency of 0.3 for allowing SNPs in palindromic regions, and *r*^2^ < 0.001 over a 10 kb region for linkage disequilibrium clumping (accounts for SNPs correlations). After the preliminary selection, Phenoscanner (12) was used to remove SNPs that potentially violate the second and third key assumptions by having pleiotropic effects on other phenotypes (body mass index, smoking status, hypertension, coronary artery disease, chronic renal failure, and diabetes). Finally, pleiotropic outliers were identified and excluded with MR pleiotropy residual sum and outlier (MR-PRESSO) (13).

### Data Sources of Outcomes

To identify genetic variants underlying AF, six discovery cohorts (HUNT, deCODE, MGI, DiscovEHR, UK Biobank, and AFGen Consortium) were gathered to carry out the GWAS of 1,030,836 people of European ancestry, including 60,620 AF cases and 970,216 controls (14) (Table 1). Phenotype-related cases in hospital, out-patient, and emergency room were collected from the electronic health record, using the International Classification of Diseases diagnosis codes (ICD, 427.3 or I48). The electrocardiogram (ECG) was digitally recorded with the Philips Page Writer cardiographs and stored in the Philips TraceMasterVue ECG Management System. Waveforms and parameters of digital ECGs were measured and adjusted for gender and age before extracting them from the database for analysis. For individuals with multiple ECG measurements, the mean standardized value was used.

### Statistical Analysis

Multiplicative random-effects inverse-variance weighted (IVW) MR analysis (15) was performed to estimate the possible association between CII and AF, presented as log odds ratios (OR) with 95% confidence intervals (CIs) per unit increase in the log OR of CD, UC, or IBD (16). Also, the methods of MR-Egger, simple median, and weighted median were employed to avoid the bias of pleiotropy effects (17). Heterogeneity was assessed with the *I*^2^ index (18) and the funnel plot (19). The power in MR analysis for each association was calculated using a web-based application (http://cnsgenomics.com/shiny/mRnd/) (20). Alpha < 0.05 was set as the two-sided threshold for statistical significance. Statistical software included STATA (StataCorp, 16) and R (R Development Core Team, 3.2.5).

### Data Availability Statements

The datasets processed in this study were acquired from GWAS (11, 14), which authorizes secondary analysis through copying and redistributing the material in any medium or format (21); thus no ethical approval was required.

## Results

**Figure 1:**
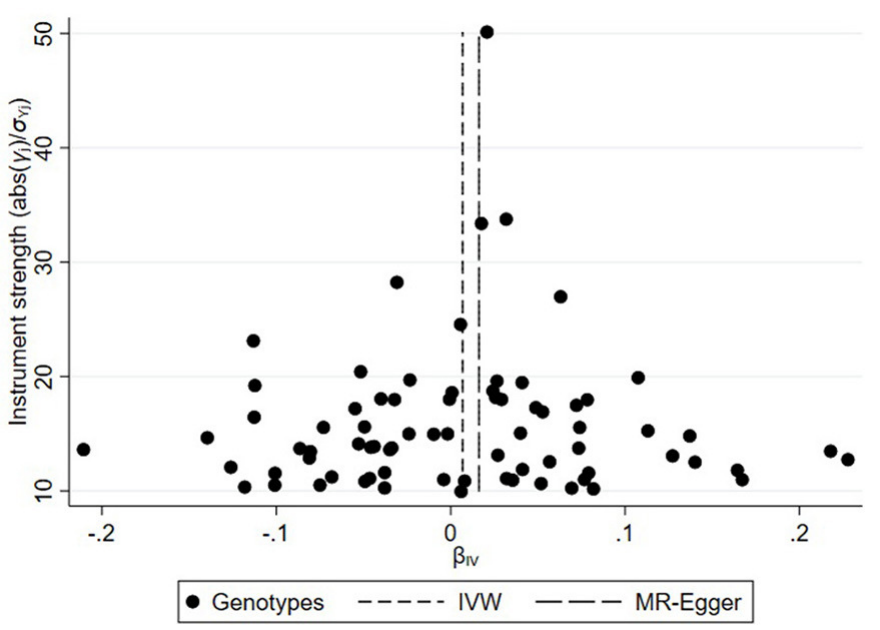
Funnel plot of genetic associations with Crohn's disease against causal estimates based on each genetic variant individually, where the causal effect is expressed in the log odds ratio of atrial fibrillation for each unit increase in Crohn's disease. The overall causal estimates (β coefficients) of Crohn's disease on atrial fibrillation given by weighted inverse-variance (short dashed line) and MR-Egger (long dashed line) methods are shown.

**Figure 2:**
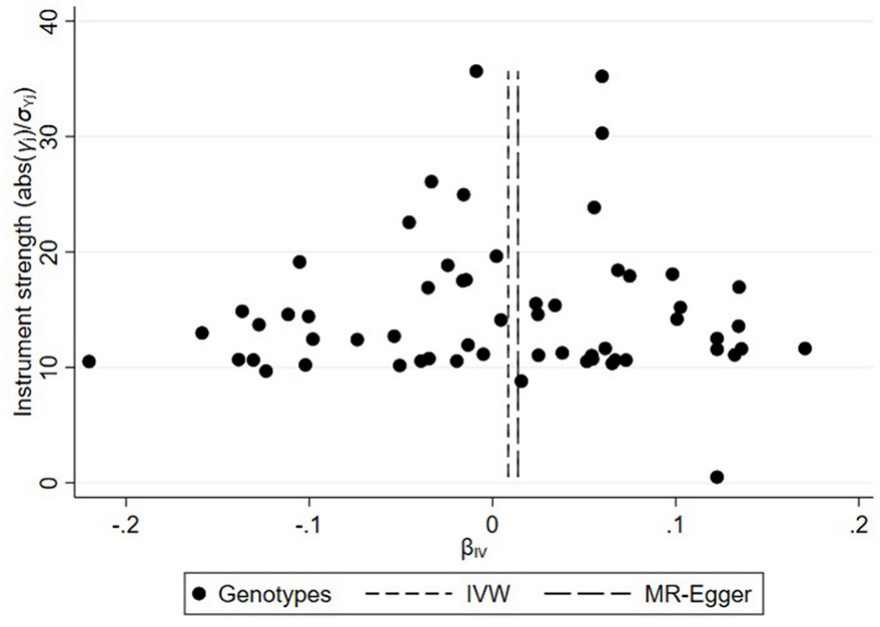
Funnel plot of genetic associations with ulcerative colitis against causal estimates based on each genetic variant individually, where the causal effect is expressed in the log odds ratio of atrial fibrillation for each unit increase in ulcerative colitis. The overall causal estimates (β coefficients) of ulcerative colitis on atrial fibrillation estimated by weighted inverse-variance (short dashed line) and MR-Egger (long dashed line) methods are shown.

On the basis of the abovementioned protocol, there were 75, 60, and 95 SNPs extracted as instrumental variables, which explained 10.0, 10.0, and 9.0% of the variance in the liability of CD, UC, and IBD, respectively. The *I*^2^ index of 30% and the funnel plots did not support substantial heterogeneity across the estimates, suggesting an absence of potential pleiotropic effects among SNPs of CD or UC (Figures 1, 2). A similar presence of low heterogeneity in IBD was displayed in Supplementary Figure 1. The detailed characteristics and associations with the remaining SNPs were presented in Supplementary Tables 1–3. The MR analysis had 75% power at an alpha rate of 5% to detect an OR of 1.08 per log odds of CD, UC, and an OR of 1.07 per log odds of IBD.

**Figure 3:**
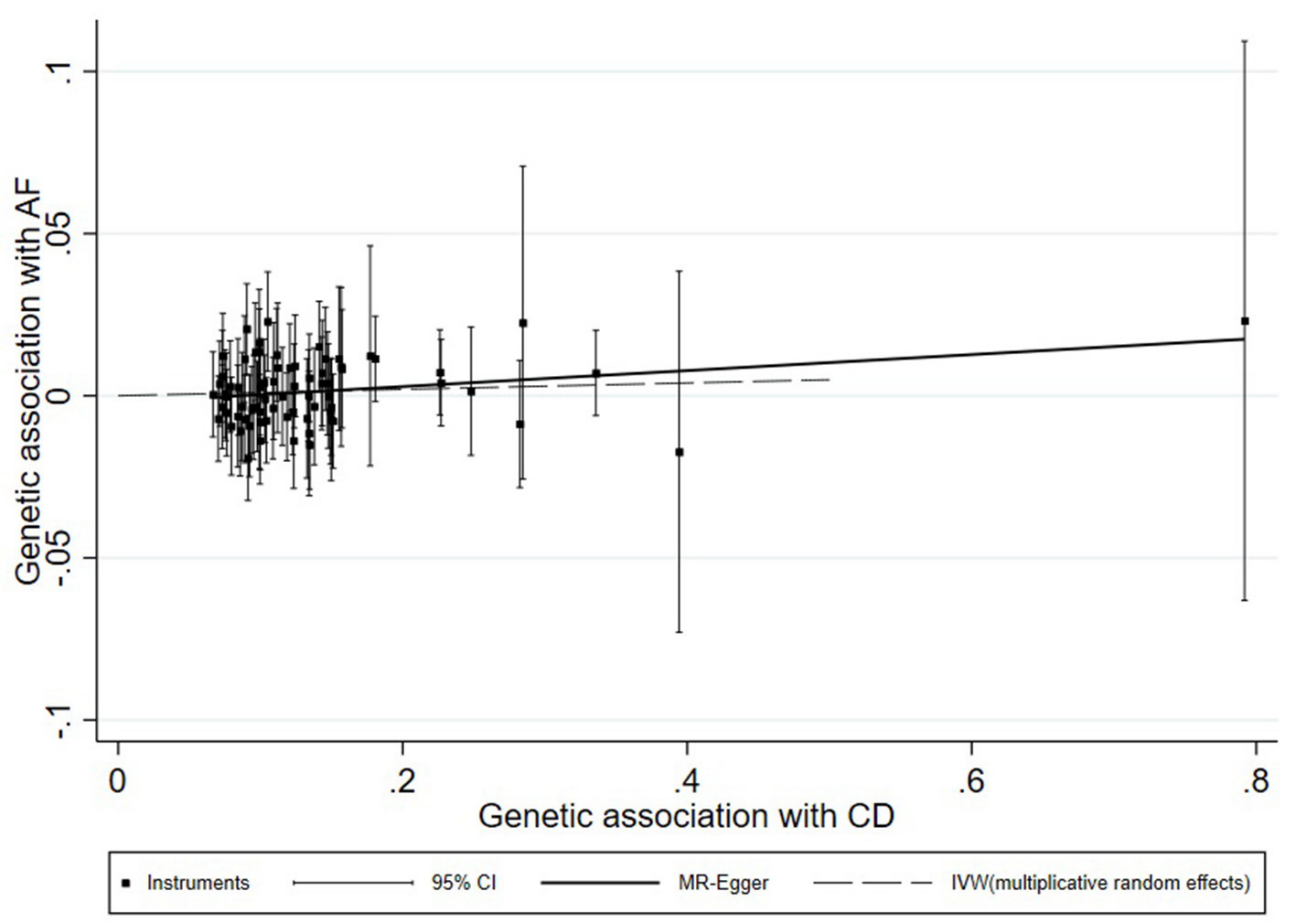
Scatterplot of genetic associations with atrial fibrillation against associations with Crohn's disease, with causal estimates (β coefficients) of Crohn's disease on atrial fibrillation estimated by weighted inverse-variance (dashed line), and MR-Egger (solid line) methods. The straight lines should be the change in the log odds of atrial fibrillation per unit increase of the log odds of Crohn's disease.

**Figure 4:**
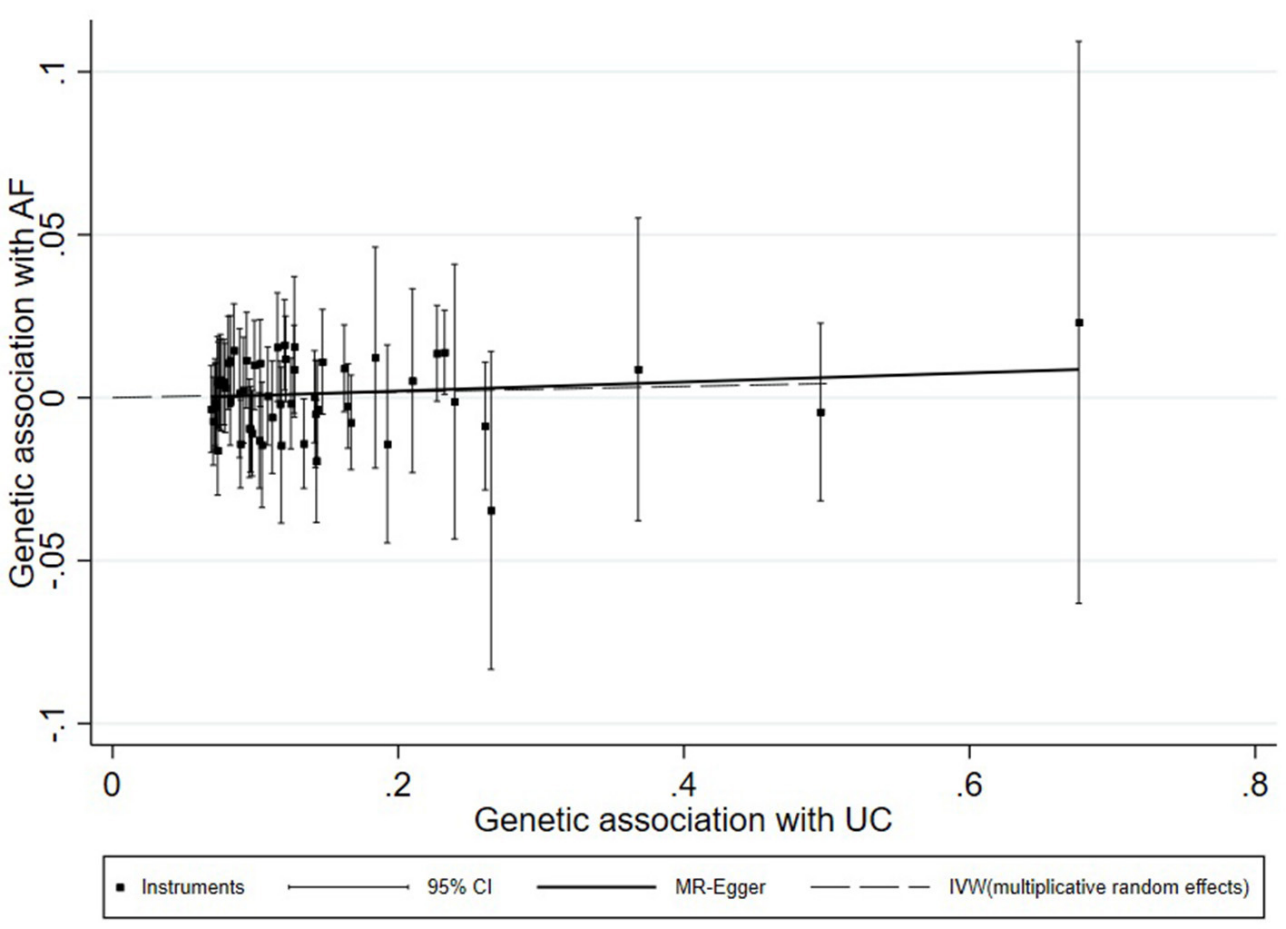
Scatterplot of genetic associations with atrial fibrillation against associations with ulcerative colitis, with causal estimates (β coefficients) of ulcerative colitis on atrial fibrillation estimated by weighted inverse-variance (dashed line), and MR-Egger (solid line) methods. The straight lines should be the change in the log odds of atrial fibrillation per unit increase of the log odds of ulcerative colitis.

A universal null association between CII and AF was captured by each multiplicative random effect IVW model (CD: OR = 1.0059, 95% CI: 0.9900, 1.0220, *p* = 0.47; UC: OR = 1.0087, 95% CI: 0.9896, 1.0281, *p* = 0.38; IBD: OR = 1.0080, 95% CI: 0.9908, 1.0255, *p* = 0.37; Table 2). Methods of MR-Egger, simple median, and the weighted median also found a lack of evidence to support a causal effect of CII on AF (Table 2). The association between each variant with CD/UC and risk of AF was displayed in Figures 3, 4. The scatterplots of IBD variants associated with AF are presented in Supplementary Figure 2. The results of leave-one-out sensitivity analysis (22) indicated that the null association was not remarkably affected by any individual SNP in each MR model (Supplementary Figures 3–5).

**Table 2:** MR estimates of the causal effect of CD, UC, and IBD on AF.

**Models**	**CD**		**UC**		**IBD**	
	**OR**	**95% CI**	**OR**	**95% CI**	**OR**	**95% CI**
Inverse variance weighted (multiplicative random effects)	1.0059	0.9900, 1.0220	1.0087	0.9896, 1.0281	1.0080	0.9908, 1.0255
MR Egger	1.0187	0.9788, 1.0603	1.0140	0.9695, 1.0605	0.9938	0.9558, 1.0333
Simple median	1.0032	0.9818, 1.0251	1.0046	0.9779, 1.0321	1.0089	0.9847, 1.0338
Weighted median	1.0189	0.9964, 1.0420	1.0004	0.9745, 1.0271	0.9936	0.9671, 1.0209

*MR, Mendelian randomization; IBD, inflammatory bowel disease; UC, ulcerative colitis; CD, Crohn's disease, AF, atrial fibrillation; OR, odds ratio; CI, confidence interval*.

## Discussion

With large-scale GWAS studies, our two-sample MR analysis demonstrated that neither CD nor UC had a causal effect on AF. With the use of IBD SNPs as the instruments, the estimated effects on AF also become null. Multiple methods including IVW, MR-Egger, the simple median, and weighted median were employed in MR analysis for each set of SNPs, which resulted in a similar conclusion, indicating the robustness of our findings.

The association between cardiac disease and CII has been reported in traditional observational studies. A nationwide investigation of IBD-associated risk of heart failure (HF) in Denmark showed that subjects with IBD had an increased risk of hospitalization for HF (23). Another population-based cohort study reported that during 13 years of follow-up after IBD diagnosis, the risk of ischemic heart disease (IHD) was increased (IRR = 1.22, 95% CI: 1.14, 1.30), as compared with IBD-free individuals (24). While focusing on arrhythmic attack, compared with age- and sex-matched controls, IBD patients corresponded to an increased AF incidence (IRR = 1.26, 95% CI: 1.16, 1.36) (25).

Despite prior reports of a potentially increased risk of cardiac disease among patients with IBD, our MR analysis did not provide sufficient evidence to support a causal role of either IBD or subcategories (CD or UC) on the risk of AF. Given the random allocation of genetic variants, MR analysis may provide a more robust result, as compared with the traditional observational study, which is more likely prone to confounders and reverse causation. The association observed in previous studies may be attributable to several common risk factors shared by IBD and AF. Cigarette smoking is a well-established and modifiable risk factor for AF (26), but it is also associated with a greater likelihood of aggressive CD including the need for surgery (27). While diabetes is a risk factor for the development of AF (28), after adjustment for steroid use and baseline glucose levels, incident diabetes also more frequently occurred in IBD patients (29). A retrospective cohort study reported that increased body weight is associated with a higher likelihood of developing CD and UC flare-ups despite ongoing medical therapy (30). A systematic review showed that general adiposity and higher body fat mass increase the risk of atrial fibrillation (31).

The large population of CD, UC, and AF patients was the main strength of this study. Compared with the association concluded by the observational study design, two-sample MR analyses warrant an exploration of causal conclusion. However, there are several limitations to the current study. While the GWAS presented the descriptive statistics of lesion location and the surgical procedures IBD patients received, these clinical characteristics related to genotype-phenotype mapping were not examined, hindering us from drawing more specific conclusions. Also, the patients' laboratory tests (biomarkers of inflammation) and flare duration were not reported in the GWAS, which could have contributed to the definition of IBD status (active vs. remission). Due to the lack of information on the monitoring and the AF identification by ECG (and not by the use of an implanted recording device), AF incidence may be underestimated, because of the nature of silent AF and cryptogenic strokes. Additionally, it is unlikely to completely remove the biased estimation of causal inference due to potential horizontal pleiotropy. However, MR-Egger regression, MR-PRESSO, and a heterogeneity test were performed, which showed no pleiotropic effect.

## Conclusions

Using two-sample MR analysis, we found no convincing evidence to support a causal effect of chronic intestinal inflammation on the risk of developing AF in a population of European ancestry.

## Data Availability Statement

Publicly available datasets were analyzed in this study. This data can be found here: https://gwas.mrcieu.ac.uk/datasets/ebi-a-GCST006414/; https://gwas.mrcieu.ac.uk/datasets/ieu-a-292/.

## Author Contributions

LC and CJ: conceptualization, data curation, and formal analysis, funding acquisition, investigation, methodology, project administration, resources, software, supervision, validation, visualization, roles/writing – original draft, and writing – review and editing. All authors contributed to the article and approved the submitted version.

## Conflict of Interest

The authors declare that the research was conducted in the absence of any commercial or financial relationships that could be construed as a potential conflict of interest.

## Supplementary Material

Supplementary Figure 1 Funnel plot of genetic associations between inflammatory bowel disease and causal estimates of atrial fibrillation.

Supplementary Figure 2 Scatterplot of genetic associations between atrial fibrillation and inflammatory bowel disease.

Supplementary Figure 3 Leave-one-out sensitivity analysis for causal effect of Crohn's disease on atrial fibrillation. The estimate is indicated by the odds ratio (OR).

Supplementary Figure 4 Leave-one-out sensitivity analysis for causal effect of ulcerative colitis on atrial fibrillation.

Supplementary Figure 5 Leave-one-out sensitivity analysis for causal effect of inflammatory bowel disease on atrial fibrillation.
