# Supplementary material for: Does Chronic Intestinal Inflammation Promote Atrial Fibrillation: A Mendelian Randomization Study With Populations of European Ancestry
Source: Front Cardiovasc Med. 2021 May 10;8:641291. doi: 10.3389/fcvm.2021.641291 (PMC8141578; doi:10.3389/fcvm.2021.641291)
Supplement: Supplementary file 1 [file Table_1.docx]

Supplemental Table S1. Characteristics of the 75 SNPs related to Crohn's Disease and Atrial Fibrillation

| SNP | Effects on Ulcerative Colitis | | | | | | Effects on Atrial fibrillation | | | | | | Chr | Position |
| --- | --- | --- | --- | --- | --- | --- | --- | --- | --- | --- | --- | --- | --- | --- |
|  | EA | OA | EAF | Beta | SE | p-val | EA | OA | EAF | Beta | SE | p-val |  |  |
| rs10185424 | G | T | 0.5396 | -0.0966 | 0.0126 | 1.47E-14 | G | T | 0.5365 | 0.0097 | 0.0067 | 0.1462 | 2 | 102662888 |
| rs10748783 | A | C | 0.5237 | -0.1648 | 0.0126 | 7.73E-39 | A | C | 0.5110 | 0.0026 | 0.0066 | 0.7003 | 10 | 101285872 |
| rs10758669 | A | C | 0.6504 | -0.1432 | 0.0129 | 1.04E-28 | A | C | 0.6473 | 0.0035 | 0.0076 | 0.6418 | 9 | 4981602 |
| rs10761659 | G | A | 0.5399 | 0.1173 | 0.0126 | 1.50E-20 | G | A | 0.5433 | -0.0019 | 0.0067 | 0.7766 | 10 | 64445564 |
| rs1077773 | A | G | 0.5238 | 0.0721 | 0.0124 | 5.96E-09 | A | G | 0.5391 | -0.0025 | 0.0067 | 0.7136 | 7 | 17442679 |
| rs11083840 | G | T | 0.4025 | 0.0691 | 0.0125 | 3.41E-08 | G | T | 0.4157 | -0.0035 | 0.0068 | 0.6114 | 19 | 47119910 |
| rs111830527 | A | G | 0.0525 | -0.1923 | 0.0293 | 5.09E-11 | A | G | 0.0520 | 0.0142 | 0.0155 | 0.3584 | 1 | 22687173 |
| rs11230563 | T | C | 0.3480 | -0.0751 | 0.0134 | 1.90E-08 | T | C | 0.3456 | -0.0041 | 0.0070 | 0.5601 | 11 | 60776209 |
| rs11641184 | A | C | 0.4762 | 0.0780 | 0.0125 | 4.24E-10 | A | C | 0.4734 | 0.0048 | 0.0067 | 0.4723 | 16 | 11704651 |
| rs12132349 | A | T | 0.2809 | -0.1669 | 0.0144 | 3.64E-31 | A | T | 0.2955 | 0.0076 | 0.0074 | 0.3056 | 1 | 200875242 |
| rs12318183 | A | C | 0.3854 | 0.1622 | 0.0127 | 1.44E-37 | A | C | 0.3947 | 0.0090 | 0.0068 | 0.1853 | 12 | 68503836 |
| rs12718244 | A | G | 0.4081 | 0.0718 | 0.0127 | 1.41E-08 | A | G | 0.4050 | -0.0028 | 0.0068 | 0.6845 | 7 | 50175654 |
| rs12796489 | A | C | 0.0229 | -0.6765 | 0.0560 | 1.22E-33 | A | C | 0.0091 | -0.0231 | 0.0440 | 0.5986 | 11 | 3059360 |
| rs13136827 | C | T | 0.1622 | -0.1118 | 0.0176 | 2.35E-10 | C | T | 0.1847 | 0.0060 | 0.0088 | 0.4988 | 4 | 123171318 |
| rs13255292 | T | C | 0.3284 | -0.0755 | 0.0137 | 3.82E-08 | T | C | 0.3265 | -0.0055 | 0.0071 | 0.4431 | 8 | 129076573 |
| rs17694108 | A | G | 0.2797 | 0.0958 | 0.0139 | 6.17E-12 | A | G | 0.2952 | -0.0094 | 0.0077 | 0.2208 | 19 | 33731551 |
| rs17780256 | C | A | 0.1927 | -0.1154 | 0.0160 | 6.13E-13 | C | A | 0.1882 | -0.0155 | 0.0085 | 0.0700 | 17 | 70642923 |
| rs2395022 | C | A | 0.9589 | -0.1839 | 0.0292 | 2.88E-10 | C | A | 0.9297 | -0.0123 | 0.0173 | 0.4794 | 7 | 98750379 |
| rs2836883 | A | G | 0.2728 | -0.2271 | 0.0147 | 1.47E-53 | A | G | 0.2794 | -0.0136 | 0.0075 | 0.0704 | 21 | 40466744 |
| rs34659678 | T | C | 0.0573 | 0.2099 | 0.0251 | 5.95E-17 | T | C | 0.0575 | 0.0052 | 0.0144 | 0.7175 | 6 | 111888540 |
| rs36070529 | A | G | 0.1999 | -0.0917 | 0.0160 | 1.04E-08 | A | G | 0.2077 | -0.0023 | 0.0083 | 0.7824 | 5 | 158619835 |
| rs3774937 | C | T | 0.3257 | 0.0993 | 0.0132 | 4.61E-14 | C | T | 0.3397 | 0.0100 | 0.0070 | 0.1566 | 4 | 103434253 |
| rs3776414 | G | T | 0.3756 | 0.0705 | 0.0128 | 4.10E-08 | G | T | 0.3613 | -0.0072 | 0.0069 | 0.2939 | 5 | 10689562 |
| rs4366152 | C | T | 0.6800 | 0.1204 | 0.0136 | 7.79E-19 | C | T | 0.6679 | 0.0162 | 0.0071 | 0.0228 | 9 | 117564875 |
| rs4456788 | A | G | 0.6106 | -0.1028 | 0.0127 | 7.07E-16 | A | G | 0.6073 | 0.0131 | 0.0075 | 0.0807 | 21 | 45616324 |
| rs4656958 | G | A | 0.6821 | 0.0824 | 0.0139 | 2.82E-09 | G | A | 0.6692 | 0.0112 | 0.0071 | 0.1152 | 1 | 160856964 |
| rs4676410 | A | G | 0.2038 | 0.1420 | 0.0157 | 1.85E-19 | A | G | 0.2131 | -0.0050 | 0.0084 | 0.5486 | 2 | 241563739 |
| rs4743820 | T | C | 0.7019 | 0.0809 | 0.0138 | 4.05E-09 | T | C | 0.6987 | 0.0107 | 0.0073 | 0.1444 | 9 | 93928416 |
| rs4747886 | T | C | 0.4081 | 0.0738 | 0.0129 | 9.58E-09 | T | C | 0.4101 | 0.0040 | 0.0067 | 0.5529 | 10 | 6176166 |
| rs4812833 | A | G | 0.5188 | 0.1033 | 0.0126 | 1.87E-16 | A | G | 0.5329 | 0.0106 | 0.0068 | 0.1182 | 20 | 43068996 |
| rs483905 | A | G | 0.2890 | 0.0850 | 0.0135 | 3.16E-10 | A | G | 0.2917 | 0.0145 | 0.0073 | 0.0470 | 11 | 96023427 |
| rs4947328 | G | A | 0.0237 | 0.2395 | 0.0381 | 3.38E-10 | G | A | 0.0333 | -0.0012 | 0.0215 | 0.9558 | 6 | 31561747 |
| rs4973341 | T | C | 0.6627 | 0.0735 | 0.0131 | 2.25E-08 | T | C | 0.6520 | 0.0048 | 0.0071 | 0.4940 | 2 | 228660362 |
| rs4976646 | C | T | 0.3415 | 0.0788 | 0.0132 | 2.52E-09 | C | T | 0.3535 | 0.0030 | 0.0070 | 0.6654 | 5 | 176788570 |
| rs55808324 | A | G | 0.0932 | 0.1272 | 0.0210 | 1.47E-09 | A | G | 0.0971 | 0.0156 | 0.0110 | 0.1574 | 14 | 88444752 |
| rs56167332 | A | C | 0.3375 | 0.1414 | 0.0132 | 7.27E-27 | A | C | 0.3509 | 0.0003 | 0.0072 | 0.9711 | 5 | 158827769 |
| rs59418206 | A | G | 0.3508 | 0.0736 | 0.0130 | 1.45E-08 | A | G | 0.3383 | -0.0162 | 0.0070 | 0.0202 | 10 | 35331624 |
| rs6111031 | T | C | 0.1591 | -0.2609 | 0.0191 | 1.33E-42 | T | C | 0.1289 | 0.0087 | 0.0100 | 0.3825 | 20 | 1682037 |
| rs61893460 | A | G | 0.4447 | 0.1211 | 0.0125 | 4.60E-22 | A | G | 0.4429 | 0.0119 | 0.0067 | 0.0745 | 11 | 76291154 |
| rs6426833 | A | G | 0.5360 | 0.2324 | 0.0126 | 3.77E-76 | A | G | 0.5181 | 0.0139 | 0.0066 | 0.0359 | 1 | 20171860 |
| rs6466198 | T | A | 0.3860 | 0.1339 | 0.0128 | 1.90E-25 | T | A | 0.3920 | -0.0141 | 0.0070 | 0.0440 | 7 | 107480126 |
| rs661054 | G | A | 0.3408 | -0.1249 | 0.0136 | 3.18E-20 | G | A | 0.3207 | 0.0018 | 0.0071 | 0.8031 | 11 | 114430410 |
| rs6920220 | A | G | 0.2086 | 0.1469 | 0.0152 | 4.78E-22 | A | G | 0.2107 | 0.0110 | 0.0082 | 0.1770 | 6 | 138006504 |
| rs7240004 | G | A | 0.3795 | -0.0824 | 0.0130 | 2.50E-10 | G | A | 0.3794 | 0.0011 | 0.0069 | 0.8758 | 18 | 46395022 |
| rs7404095 | C | T | 0.5796 | 0.0718 | 0.0127 | 1.52E-08 | C | T | 0.5887 | -0.0014 | 0.0068 | 0.8336 | 16 | 23864590 |
| rs7547569 | C | T | 0.0668 | -0.4957 | 0.0292 | 8.71E-65 | C | T | 0.0653 | 0.0044 | 0.0139 | 0.7534 | 1 | 67731368 |
| rs7608910 | G | A | 0.3909 | 0.1271 | 0.0127 | 1.25E-23 | G | A | 0.3655 | 0.0087 | 0.0069 | 0.2064 | 2 | 61204856 |
| rs76546301 | A | G | 0.0182 | 0.2650 | 0.0410 | 1.05E-10 | A | G | 0.0183 | -0.0346 | 0.0249 | 0.1648 | 7 | 50498389 |
| rs76904798 | T | C | 0.1368 | 0.1046 | 0.0176 | 2.78E-09 | T | C | 0.1344 | -0.0145 | 0.0098 | 0.1387 | 12 | 40614434 |
| rs7711427 | C | A | 0.6130 | 0.0889 | 0.0128 | 3.67E-12 | C | A | 0.6037 | 0.0014 | 0.0101 | 0.8870 | 5 | 40414886 |
| rs7738430 | C | T | 0.0262 | 0.3680 | 0.0341 | 3.51E-27 | C | T | 0.0222 | 0.0087 | 0.0237 | 0.7139 | 6 | 31508836 |
| rs79045992 | A | G | 0.1033 | 0.1181 | 0.0208 | 1.43E-08 | A | G | 0.1095 | -0.0146 | 0.0122 | 0.2327 | 16 | 68518992 |
| rs8096327 | G | A | 0.3839 | 0.0938 | 0.0128 | 2.24E-13 | A | T | 0.0016 | 0.2038 | 0.1891 | 0.2813 | 18 | 12887750 |
| rs8096327 | G | A | 0.3839 | 0.0938 | 0.0128 | 2.24E-13 | G | A | 0.3956 | 0.0115 | 0.1891 | 0.2813 | 18 | 12887750 |
| rs8096327 | G | A | 0.3839 | 0.0938 | 0.0128 | 2.24E-13 | G | A | 0.3956 | 0.0115 | 0.0075 | 0.1250 | 18 | 12887750 |
| rs913678 | C | T | 0.3293 | -0.0758 | 0.0133 | 1.23E-08 | C | T | 0.3322 | -0.0039 | 0.0072 | 0.5932 | 20 | 48955424 |
| rs941823 | C | T | 0.7509 | 0.1087 | 0.0147 | 1.39E-13 | C | T | 0.7326 | 0.0005 | 0.0077 | 0.9530 | 13 | 41013977 |
| rs9611131 | C | T | 0.1477 | -0.1427 | 0.0182 | 3.84E-15 | C | T | 0.1424 | 0.0195 | 0.0096 | 0.0429 | 22 | 39662480 |
| rs9891119 | C | A | 0.3536 | -0.0895 | 0.0133 | 1.72E-11 | C | A | 0.3619 | 0.0142 | 0.0069 | 0.0394 | 17 | 40507980 |
| rs9941524 | G | A | 0.4559 | 0.0977 | 0.0128 | 2.15E-14 | G | A | 0.4425 | -0.0109 | 0.0067 | 0.1070 | 2 | 199499443 |

Abbreviation: EA, Effect Allele; OA, Other Allele; EAF, effect allele frequency; SE, standard error; SNP, single nucleotide polymorphism; Chr, Chromosome
