# Supplementary material for: Does Chronic Intestinal Inflammation Promote Atrial Fibrillation: A Mendelian Randomization Study With Populations of European Ancestry
Source: Front Cardiovasc Med. 2021 May 10;8:641291. doi: 10.3389/fcvm.2021.641291 (PMC8141578; doi:10.3389/fcvm.2021.641291)
Supplement: Supplementary file 2 [file Table_2.docx]

Supplemental Table S2. Characteristics of the 60 SNPs related to Ulcerative Colitis and Atrial Fibrillation

| SNP | Effects on Ulcerative Colitis | | | | | | Effects on Atrial fibrillation | | | | | | Chr | Position |
| --- | --- | --- | --- | --- | --- | --- | --- | --- | --- | --- | --- | --- | --- | --- |
|  | EA | OA | EAF | Beta | SE | p-val | EA | OA | EAF | Beta | SE | p-val |  |  |
| rs10758669 | A | C | 0.6504 | -0.1498 | 0.0123 | 4.19E-34 | A | C | 0.6473 | 0.0035 | 0.0076 | 0.6418 | 9 | 4981602 |
| rs10798069 | T | G | 0.4925 | -0.0704 | 0.0120 | 4.25E-09 | T | G | 0.5047 | 0.0071 | 0.0067 | 0.2861 | 1 | 186875459 |
| rs10800309 | G | A | 0.6578 | -0.0904 | 0.0126 | 8.48E-13 | G | A | 0.6715 | -0.0206 | 0.0071 | 0.0038 | 1 | 161472158 |
| rs10878302 | A | T | 0.9286 | 0.1568 | 0.0238 | 4.20E-11 | A | T | 0.9242 | 0.0089 | 0.0125 | 0.4763 | 12 | 40669826 |
| rs10956252 | G | C | 0.6189 | 0.1186 | 0.0124 | 8.34E-22 | G | C | 0.6170 | -0.0065 | 0.0069 | 0.3431 | 8 | 126536137 |
| rs11117431 | G | A | 0.1978 | -0.1493 | 0.0164 | 1.09E-19 | G | A | 0.2135 | 0.0048 | 0.0083 | 0.5660 | 16 | 86015316 |
| rs11152949 | G | A | 0.3195 | 0.1338 | 0.0129 | 2.18E-25 | G | A | 0.3237 | 0.0001 | 0.0072 | 0.9870 | 6 | 106449085 |
| rs11159833 | T | C | 0.0868 | 0.1552 | 0.0208 | 7.59E-14 | T | C | 0.0913 | 0.0114 | 0.0113 | 0.3154 | 14 | 88476004 |
| rs11236797 | A | C | 0.4444 | 0.1807 | 0.0121 | 8.54E-51 | A | C | 0.4424 | 0.0114 | 0.0067 | 0.0891 | 11 | 76299649 |
| rs11713774 | C | T | 0.1427 | 0.1327 | 0.0172 | 1.09E-14 | C | T | 0.1496 | -0.0070 | 0.0094 | 0.4546 | 3 | 18765978 |
| rs12411259 | A | G | 0.2401 | 0.1344 | 0.0137 | 1.43E-22 | A | G | 0.2358 | 0.0055 | 0.0069 | 0.4195 | 1 | 172866210 |
| rs1267501 | C | T | 0.8106 | 0.0872 | 0.0152 | 9.69E-09 | C | T | 0.7939 | -0.0033 | 0.0085 | 0.6978 | 6 | 14715257 |
| rs12694846 | G | A | 0.2593 | 0.1154 | 0.0136 | 2.50E-17 | G | A | 0.2613 | -0.0002 | 0.0077 | 0.9798 | 2 | 231148128 |
| rs12796489 | A | C | 0.0229 | -0.7918 | 0.0527 | 4.96E-51 | A | C | 0.0091 | -0.0231 | 0.0440 | 0.5986 | 11 | 3059360 |
| rs1292053 | G | A | 0.4420 | 0.0912 | 0.0119 | 1.75E-14 | G | A | 0.4415 | -0.0192 | 0.0067 | 0.0039 | 17 | 57963537 |
| rs12949918 | C | T | 0.4185 | -0.1042 | 0.0124 | 3.47E-17 | C | T | 0.4372 | 0.0076 | 0.0067 | 0.2527 | 17 | 40526273 |
| rs13001325 | T | C | 0.3757 | -0.1227 | 0.0126 | 1.68E-22 | T | C | 0.3865 | 0.0049 | 0.0068 | 0.4689 | 2 | 102939036 |
| rs1456896 | T | C | 0.6894 | 0.0977 | 0.0131 | 1.03E-13 | T | C | 0.6607 | -0.0033 | 0.0071 | 0.6444 | 7 | 50304461 |
| rs1569328 | T | C | 0.1702 | -0.1092 | 0.0167 | 6.47E-11 | T | C | 0.1611 | -0.0045 | 0.0092 | 0.6208 | 14 | 75741751 |
| rs1646019 | T | C | 0.3030 | -0.1114 | 0.0134 | 8.62E-17 | T | C | 0.2910 | -0.0126 | 0.0073 | 0.0822 | 16 | 11359680 |
| rs17129991 | T | C | 0.0225 | -0.2845 | 0.0451 | 2.81E-10 | T | C | 0.0233 | -0.0225 | 0.0246 | 0.3607 | 1 | 67862986 |
| rs17388425 | G | C | 0.1598 | -0.1573 | 0.0172 | 6.54E-20 | G | C | 0.1764 | -0.0083 | 0.0093 | 0.3704 | 5 | 158824174 |
| rs17694108 | A | G | 0.2797 | 0.0796 | 0.0135 | 3.29E-09 | A | G | 0.2952 | -0.0094 | 0.0077 | 0.2208 | 19 | 33731551 |
| rs181826 | A | C | 0.6266 | 0.0996 | 0.0127 | 4.53E-15 | A | C | 0.6155 | -0.0139 | 0.0068 | 0.0416 | 5 | 141526057 |
| rs1927681 | A | T | 0.4430 | 0.0888 | 0.0121 | 2.42E-13 | A | T | 0.4429 | 0.0113 | 0.0068 | 0.0960 | 13 | 27558881 |
| rs2024092 | A | G | 0.2162 | 0.1477 | 0.0143 | 7.13E-25 | A | G | 0.2046 | -0.0001 | 0.0082 | 0.9923 | 19 | 1124031 |
| rs212388 | T | C | 0.6043 | -0.1024 | 0.0124 | 1.80E-16 | T | C | 0.5864 | -0.0041 | 0.0068 | 0.5485 | 6 | 159490436 |
| rs2153283 | A | C | 0.2170 | -0.1088 | 0.0155 | 2.39E-12 | A | C | 0.2138 | 0.0038 | 0.0080 | 0.6313 | 10 | 59972299 |
| rs2227551 | T | G | 0.7288 | 0.0994 | 0.0137 | 4.72E-13 | T | G | 0.7042 | -0.0080 | 0.0074 | 0.2789 | 10 | 75669190 |
| rs2270395 | T | C | 0.7616 | 0.1243 | 0.0145 | 8.93E-18 | T | C | 0.7581 | 0.0092 | 0.0080 | 0.2537 | 16 | 50846832 |
| rs2284553 | G | A | 0.5904 | 0.1032 | 0.0123 | 5.63E-17 | G | A | 0.5832 | -0.0010 | 0.0069 | 0.8872 | 21 | 34776695 |
| rs2395022 | C | A | 0.9589 | -0.1772 | 0.0282 | 3.13E-10 | C | A | 0.9297 | -0.0123 | 0.0173 | 0.4794 | 7 | 98750379 |
| rs2413583 | T | C | 0.1654 | -0.2104 | 0.0168 | 7.72E-36 | T | C | 0.1590 | 0.0238 | 0.0091 | 0.0090 | 22 | 39659773 |
| rs2538470 | G | A | 0.6378 | -0.0750 | 0.0123 | 1.05E-09 | G | A | 0.6254 | -0.0006 | 0.0069 | 0.9319 | 7 | 148220448 |
| rs259964 | G | A | 0.5414 | -0.0713 | 0.0119 | 2.08E-09 | G | A | 0.5428 | -0.0037 | 0.0067 | 0.5775 | 20 | 57824309 |
| rs28999107 | T | G | 0.4387 | 0.0857 | 0.0127 | 1.29E-11 | T | G | 0.4460 | -0.0108 | 0.0071 | 0.1269 | 12 | 6493100 |
| rs303429 | T | C | 0.5997 | 0.0763 | 0.0124 | 8.38E-10 | T | C | 0.5933 | -0.0052 | 0.0068 | 0.4442 | 10 | 30708441 |
| rs34779708 | G | T | 0.3512 | 0.1345 | 0.0124 | 1.90E-27 | G | T | 0.3394 | -0.0151 | 0.0070 | 0.0299 | 10 | 35466185 |
| rs34787213 | T | C | 0.1401 | -0.1497 | 0.0183 | 2.85E-16 | T | C | 0.1373 | 0.0074 | 0.0096 | 0.4409 | 11 | 60799046 |
| rs34804116 | A | C | 0.3867 | -0.0939 | 0.0127 | 1.27E-13 | A | C | 0.3976 | 0.0043 | 0.0068 | 0.5262 | 5 | 72539850 |
| rs35164067 | A | G | 0.2039 | -0.1434 | 0.0156 | 3.19E-20 | A | G | 0.1998 | -0.0070 | 0.0083 | 0.3980 | 19 | 10525181 |
| rs35320439 | C | T | 0.3104 | 0.0841 | 0.0138 | 9.89E-10 | C | T | 0.3132 | -0.0063 | 0.0080 | 0.4303 | 2 | 242737341 |
| rs35730213 | C | G | 0.2807 | -0.1511 | 0.0138 | 7.84E-28 | C | G | 0.2955 | 0.0078 | 0.0074 | 0.2901 | 1 | 200874229 |
| rs3776414 | G | T | 0.3756 | 0.0888 | 0.0123 | 5.04E-13 | G | T | 0.3613 | -0.0072 | 0.0069 | 0.2939 | 5 | 10689562 |
| rs3801810 | A | G | 0.2337 | 0.1051 | 0.0140 | 6.63E-14 | A | G | 0.2420 | 0.0229 | 0.0078 | 0.0032 | 7 | 26892531 |
| rs4703855 | T | C | 0.2998 | -0.0733 | 0.0132 | 3.03E-08 | T | C | 0.3002 | -0.0060 | 0.0072 | 0.4050 | 5 | 71693899 |
| rs559928 | C | T | 0.8128 | 0.0991 | 0.0158 | 3.75E-10 | C | T | 0.7930 | 0.0163 | 0.0084 | 0.0528 | 11 | 64150370 |
| rs6074022 | T | C | 0.7497 | -0.0963 | 0.0138 | 2.70E-12 | T | C | 0.7364 | -0.0135 | 0.0077 | 0.0784 | 20 | 44740196 |
| rs6111031 | T | C | 0.1591 | -0.2824 | 0.0181 | 9.61E-55 | T | C | 0.1289 | 0.0087 | 0.0100 | 0.3825 | 20 | 1682037 |
| rs640466 | C | T | 0.3739 | -0.0758 | 0.0125 | 1.31E-09 | C | T | 0.3812 | 0.0003 | 0.0069 | 0.9612 | 19 | 34670725 |
| rs6456426 | A | C | 0.4984 | -0.0992 | 0.0120 | 1.37E-16 | A | C | 0.4949 | -0.0136 | 0.0067 | 0.0424 | 6 | 21438889 |
| rs6500315 | G | A | 0.7753 | 0.1455 | 0.0146 | 2.18E-23 | G | A | 0.7557 | 0.0114 | 0.0081 | 0.1585 | 16 | 50508101 |
| rs6561151 | A | G | 0.2235 | 0.1471 | 0.0142 | 4.68E-25 | A | G | 0.2048 | 0.0038 | 0.0081 | 0.6379 | 13 | 44484706 |
| rs6738490 | C | T | 0.5274 | 0.2262 | 0.0121 | 4.26E-78 | C | T | 0.5198 | 0.0072 | 0.0067 | 0.2785 | 2 | 234161583 |
| rs6740462 | A | C | 0.7378 | 0.0997 | 0.0141 | 1.74E-12 | A | C | 0.7245 | 0.0027 | 0.0076 | 0.7198 | 2 | 65667272 |
| rs7015630 | C | T | 0.2657 | -0.0842 | 0.0138 | 9.00E-10 | C | T | 0.2660 | -0.0027 | 0.0076 | 0.7230 | 8 | 90875918 |
| rs71624119 | A | G | 0.2422 | -0.0923 | 0.0149 | 6.57E-10 | A | G | 0.2490 | 0.0093 | 0.0080 | 0.2463 | 5 | 55440730 |
| rs7194886 | T | C | 0.4357 | -0.2270 | 0.0122 | 1.42E-77 | T | C | 0.4275 | -0.0040 | 0.0068 | 0.5513 | 16 | 50725193 |
| rs7236492 | T | C | 0.1537 | -0.0997 | 0.0173 | 9.09E-09 | T | C | 0.1655 | 0.0049 | 0.0092 | 0.5950 | 18 | 77220616 |
| rs7517847 | G | T | 0.4352 | -0.3358 | 0.0125 | 1.38E-159 | G | T | 0.4590 | -0.0070 | 0.0067 | 0.2917 | 1 | 67681669 |
| rs7608910 | G | A | 0.3909 | 0.1206 | 0.0121 | 2.95E-23 | G | A | 0.3655 | 0.0087 | 0.0069 | 0.2064 | 2 | 61204856 |
| rs76906269 | G | A | 0.0188 | 0.3943 | 0.0370 | 1.75E-26 | G | A | 0.0164 | -0.0173 | 0.0284 | 0.5436 | 12 | 40607709 |
| rs7711427 | C | A | 0.6130 | 0.2480 | 0.0125 | 5.17E-88 | C | A | 0.6037 | 0.0014 | 0.0101 | 0.8870 | 5 | 40414886 |
| rs7773324 | A | G | 0.6002 | 0.0787 | 0.0129 | 1.06E-09 | A | G | 0.6017 | 0.0028 | 0.0072 | 0.6980 | 6 | 382559 |
| rs7786444 | T | C | 0.1163 | 0.1121 | 0.0183 | 9.83E-10 | T | C | 0.1170 | 0.0086 | 0.0102 | 0.4029 | 7 | 28154384 |
| rs7848647 | C | T | 0.6746 | 0.1413 | 0.0130 | 1.55E-27 | C | T | 0.6631 | 0.0152 | 0.0071 | 0.0327 | 9 | 117569046 |
| rs7969592 | G | A | 0.4746 | -0.0732 | 0.0120 | 1.04E-09 | G | A | 0.4660 | 0.0034 | 0.0066 | 0.6062 | 12 | 68579649 |
| rs79980175 | C | A | 0.1360 | -0.1343 | 0.0182 | 1.70E-13 | C | A | 0.1305 | 0.0116 | 0.0098 | 0.2359 | 5 | 40521892 |
| rs8127691 | C | T | 0.6132 | -0.1234 | 0.0122 | 4.48E-24 | C | T | 0.6090 | 0.0139 | 0.0075 | 0.0640 | 21 | 45614860 |
| rs915286 | A | G | 0.5488 | 0.0666 | 0.0120 | 2.59E-08 | A | G | 0.5464 | 0.0004 | 0.0067 | 0.9463 | 13 | 40695992 |
| rs9457247 | T | C | 0.5398 | 0.1237 | 0.0124 | 2.08E-23 | T | C | 0.5273 | 0.0030 | 0.0066 | 0.6542 | 6 | 167392174 |
| rs9491892 | G | T | 0.1496 | 0.1379 | 0.0164 | 3.80E-17 | G | T | 0.1567 | -0.0033 | 0.0092 | 0.7191 | 6 | 128280358 |
| rs9554587 | G | A | 0.2242 | -0.0952 | 0.0147 | 8.29E-11 | G | A | 0.2102 | 0.0036 | 0.0082 | 0.6599 | 13 | 100040654 |
| rs9594766 | A | G | 0.5294 | -0.0736 | 0.0121 | 1.39E-09 | A | G | 0.5548 | -0.0123 | 0.0067 | 0.0651 | 13 | 43040043 |
| rs9889296 | A | G | 0.2723 | -0.1431 | 0.0138 | 2.96E-25 | A | G | 0.2938 | -0.0038 | 0.0073 | 0.6060 | 17 | 32570547 |

Abbreviation: EA, Effect Allele; OA, Other Allele; EAF, effect allele frequency; SE, standard error; SNP, single nucleotide polymorphism; Chr, Chromosome
