# Supplementary material for: Does Chronic Intestinal Inflammation Promote Atrial Fibrillation: A Mendelian Randomization Study With Populations of European Ancestry
Source: Front Cardiovasc Med. 2021 May 10;8:641291. doi: 10.3389/fcvm.2021.641291 (PMC8141578; doi:10.3389/fcvm.2021.641291)
Supplement: Supplementary file 3 [file Table_3.docx]

Supplemental Table S3. Characteristics of the 95 SNPs related to Inflammatory Bowel Disease and Atrial Fibrillation

| SNP | Effects on Ulcerative Colitis | | | | | | Effects on Atrial fibrillation | | | | | | Chr | Position |
| --- | --- | --- | --- | --- | --- | --- | --- | --- | --- | --- | --- | --- | --- | --- |
|  | EA | OA | EAF | Beta | SE | p-val | EA | OA | EAF | Beta | SE | p-val |  |  |
| rs10142466 | G | A | 0.5065 | -0.0580 | 0.0101 | 1.08E-08 | G | A | 0.5017 | -0.0091 | 0.0066 | 0.1713 | 14 | 69271784 |
| rs10758669 | A | C | 0.6504 | -0.1488 | 0.0102 | 4.70E-48 | A | C | 0.6473 | 0.0035 | 0.0076 | 0.6418 | 9 | 4981602 |
| rs10761659 | G | A | 0.5399 | 0.1538 | 0.0100 | 4.97E-53 | G | A | 0.5433 | -0.0019 | 0.0067 | 0.7766 | 10 | 64445564 |
| rs10800309 | G | A | 0.6578 | -0.1321 | 0.0104 | 6.15E-37 | G | A | 0.6715 | -0.0206 | 0.0071 | 0.0038 | 1 | 161472158 |
| rs10878302 | A | T | 0.9286 | 0.1124 | 0.0193 | 5.26E-09 | A | T | 0.9242 | 0.0089 | 0.0125 | 0.4763 | 12 | 40669826 |
| rs10956252 | G | C | 0.6189 | 0.0835 | 0.0102 | 2.26E-16 | G | C | 0.6170 | -0.0065 | 0.0069 | 0.3431 | 8 | 126536137 |
| rs11152949 | G | A | 0.3195 | 0.1051 | 0.0107 | 7.25E-23 | G | A | 0.3237 | 0.0001 | 0.0072 | 0.9870 | 6 | 106449085 |
| rs11230563 | T | C | 0.3480 | -0.0812 | 0.0106 | 1.71E-14 | T | C | 0.3456 | -0.0041 | 0.0070 | 0.5601 | 11 | 60776209 |
| rs11236797 | A | C | 0.4444 | 0.1509 | 0.0100 | 9.32E-52 | A | C | 0.4424 | 0.0114 | 0.0067 | 0.0891 | 11 | 76299649 |
| rs11641016 | G | C | 0.1973 | -0.1113 | 0.0134 | 9.51E-17 | G | C | 0.2133 | 0.0047 | 0.0083 | 0.5734 | 16 | 86014881 |
| rs11713774 | C | T | 0.1427 | 0.0943 | 0.0143 | 3.92E-11 | C | T | 0.1496 | -0.0070 | 0.0094 | 0.4546 | 3 | 18765978 |
| rs11758694 | A | T | 0.0994 | 0.1078 | 0.0160 | 1.75E-11 | A | T | 0.0815 | 0.0056 | 0.0163 | 0.7314 | 6 | 111948621 |
| rs12318183 | A | C | 0.3854 | 0.1095 | 0.0101 | 1.67E-27 | A | C | 0.3947 | 0.0090 | 0.0068 | 0.1853 | 12 | 68503836 |
| rs12411259 | A | G | 0.2401 | 0.0669 | 0.0115 | 6.18E-09 | A | G | 0.2358 | 0.0055 | 0.0069 | 0.4195 | 1 | 172866210 |
| rs1250566 | A | G | 0.3161 | -0.1009 | 0.0110 | 4.77E-20 | A | G | 0.3321 | 0.0209 | 0.0071 | 0.0034 | 10 | 81046453 |
| rs12585310 | A | G | 0.3136 | 0.0706 | 0.0108 | 5.25E-11 | A | G | 0.3075 | 0.0145 | 0.0073 | 0.0470 | 13 | 27528347 |
| rs1267499 | C | T | 0.8100 | 0.0821 | 0.0125 | 5.22E-11 | C | T | 0.7933 | -0.0028 | 0.0085 | 0.7440 | 6 | 14715882 |
| rs12718244 | A | G | 0.4081 | 0.0762 | 0.0100 | 3.35E-14 | A | G | 0.4050 | -0.0028 | 0.0068 | 0.6845 | 7 | 50175654 |
| rs12722515 | A | C | 0.1627 | -0.0989 | 0.0143 | 4.57E-12 | A | C | 0.1860 | -0.0128 | 0.0087 | 0.1413 | 10 | 6081230 |
| rs12796489 | A | C | 0.0229 | -0.7604 | 0.0432 | 2.87E-69 | A | C | 0.0091 | -0.0231 | 0.0440 | 0.5986 | 11 | 3059360 |
| rs1292053 | G | A | 0.4420 | 0.0701 | 0.0098 | 9.89E-13 | G | A | 0.4415 | -0.0192 | 0.0067 | 0.0039 | 17 | 57963537 |
| rs13107612 | T | C | 0.2969 | 0.0733 | 0.0109 | 1.62E-11 | T | C | 0.2909 | 0.0086 | 0.0073 | 0.2379 | 4 | 102739980 |
| rs13204742 | T | G | 0.1267 | 0.0916 | 0.0148 | 5.39E-10 | T | G | 0.1300 | -0.0032 | 0.0101 | 0.7497 | 6 | 128245765 |
| rs1388585 | A | G | 0.9808 | -0.3049 | 0.0317 | 6.85E-22 | A | G | 0.9492 | 0.0278 | 0.0265 | 0.2946 | 12 | 40531691 |
| rs1420098 | C | T | 0.3877 | -0.0953 | 0.0103 | 1.83E-20 | C | T | 0.3958 | 0.0030 | 0.0068 | 0.6568 | 2 | 102984279 |
| rs1517352 | C | A | 0.6048 | 0.0779 | 0.0103 | 3.87E-14 | C | A | 0.6094 | -0.0029 | 0.0068 | 0.6695 | 2 | 191931464 |
| rs1569328 | T | C | 0.1702 | -0.0810 | 0.0137 | 3.21E-09 | T | C | 0.1611 | -0.0045 | 0.0092 | 0.6208 | 14 | 75741751 |
| rs17651741 | A | G | 0.1910 | 0.0702 | 0.0127 | 2.81E-08 | A | G | 0.1857 | 0.0074 | 0.0085 | 0.3875 | 15 | 38869666 |
| rs17694108 | A | G | 0.2797 | 0.0858 | 0.0111 | 1.21E-14 | A | G | 0.2952 | -0.0094 | 0.0077 | 0.2208 | 19 | 33731551 |
| rs17780256 | C | A | 0.1927 | -0.0834 | 0.0126 | 3.19E-11 | C | A | 0.1882 | -0.0155 | 0.0085 | 0.0700 | 17 | 70642923 |
| rs181826 | A | C | 0.6266 | 0.0820 | 0.0104 | 4.05E-15 | A | C | 0.6155 | -0.0139 | 0.0068 | 0.0416 | 5 | 141526057 |
| rs1847472 | A | C | 0.3421 | -0.0673 | 0.0109 | 6.63E-10 | A | C | 0.3485 | -0.0016 | 0.0070 | 0.8197 | 6 | 90973159 |
| rs2024092 | A | G | 0.2162 | 0.1068 | 0.0121 | 1.12E-18 | A | G | 0.2046 | -0.0001 | 0.0082 | 0.9923 | 19 | 1124031 |
| rs2050392 | A | G | 0.6003 | 0.0691 | 0.0103 | 1.87E-11 | A | G | 0.5972 | -0.0058 | 0.0070 | 0.4069 | 10 | 30691503 |
| rs2143178 | C | T | 0.1658 | -0.1767 | 0.0137 | 4.80E-38 | C | T | 0.1590 | 0.0267 | 0.0091 | 0.0034 | 22 | 39660829 |
| rs2153283 | A | C | 0.2170 | -0.0860 | 0.0127 | 1.54E-11 | A | C | 0.2138 | 0.0038 | 0.0080 | 0.6313 | 10 | 59972299 |
| rs2270395 | T | C | 0.7616 | 0.0778 | 0.0119 | 5.17E-11 | T | C | 0.7581 | 0.0092 | 0.0080 | 0.2537 | 16 | 50846832 |
| rs2297559 | A | G | 0.6822 | 0.0742 | 0.0110 | 1.88E-11 | A | G | 0.6693 | 0.0110 | 0.0071 | 0.1214 | 1 | 160854526 |
| rs2395022 | C | A | 0.9589 | -0.1816 | 0.0234 | 8.27E-15 | C | A | 0.9297 | -0.0123 | 0.0173 | 0.4794 | 7 | 98750379 |
| rs2538470 | G | A | 0.6378 | -0.0676 | 0.0102 | 3.00E-11 | G | A | 0.6254 | -0.0006 | 0.0069 | 0.9319 | 7 | 148220448 |
| rs259964 | G | A | 0.5414 | -0.0675 | 0.0098 | 6.93E-12 | G | A | 0.5428 | -0.0037 | 0.0067 | 0.5775 | 20 | 57824309 |
| rs2836883 | A | G | 0.2728 | -0.1684 | 0.0115 | 3.38E-48 | A | G | 0.2794 | -0.0136 | 0.0075 | 0.0704 | 21 | 40466744 |
| rs2847278 | T | C | 0.8410 | -0.1445 | 0.0132 | 8.33E-28 | T | C | 0.8073 | 0.0037 | 0.0088 | 0.6758 | 18 | 12778715 |
| rs34779708 | G | T | 0.3512 | 0.1067 | 0.0102 | 2.07E-25 | G | T | 0.3394 | -0.0151 | 0.0070 | 0.0299 | 10 | 35466185 |
| rs34804116 | A | C | 0.3867 | -0.0575 | 0.0104 | 3.62E-08 | A | C | 0.3976 | 0.0043 | 0.0068 | 0.5262 | 5 | 72539850 |
| rs35164067 | A | G | 0.2039 | -0.1175 | 0.0127 | 2.66E-20 | A | G | 0.1998 | -0.0070 | 0.0083 | 0.3980 | 19 | 10525181 |
| rs35256947 | C | T | 0.2594 | 0.0822 | 0.0113 | 3.87E-13 | C | T | 0.2614 | -0.0009 | 0.0077 | 0.9063 | 2 | 231161026 |
| rs35730213 | C | G | 0.2807 | -0.1596 | 0.0114 | 8.33E-45 | C | G | 0.2955 | 0.0078 | 0.0074 | 0.2901 | 1 | 200874229 |
| rs36048684 | A | T | 0.1107 | -0.0941 | 0.0160 | 3.70E-09 | A | T | 0.1101 | 0.0103 | 0.0108 | 0.3389 | 5 | 158822929 |
| rs367569 | T | C | 0.2891 | -0.0958 | 0.0113 | 1.93E-17 | T | C | 0.2799 | -0.0153 | 0.0074 | 0.0381 | 16 | 11365500 |
| rs3776414 | G | T | 0.3756 | 0.0774 | 0.0102 | 2.65E-14 | G | T | 0.3613 | -0.0072 | 0.0069 | 0.2939 | 5 | 10689562 |
| rs3801835 | T | C | 0.3447 | 0.0641 | 0.0106 | 1.47E-09 | T | C | 0.3411 | 0.0058 | 0.0070 | 0.4051 | 7 | 26852053 |
| rs4692386 | C | T | 0.5930 | 0.0580 | 0.0102 | 1.21E-08 | C | T | 0.5838 | 0.0031 | 0.0069 | 0.6538 | 4 | 26132361 |
| rs4703855 | T | C | 0.2998 | -0.0711 | 0.0109 | 7.16E-11 | T | C | 0.3002 | -0.0060 | 0.0072 | 0.4050 | 5 | 71693899 |
| rs4743820 | T | C | 0.7019 | 0.0640 | 0.0109 | 3.80E-09 | T | C | 0.6987 | 0.0107 | 0.0073 | 0.1444 | 9 | 93928416 |
| rs55808324 | A | G | 0.0932 | 0.1412 | 0.0168 | 5.13E-17 | A | G | 0.0971 | 0.0156 | 0.0110 | 0.1574 | 14 | 88444752 |
| rs559928 | C | T | 0.8128 | 0.0944 | 0.0130 | 3.33E-13 | C | T | 0.7930 | 0.0163 | 0.0084 | 0.0528 | 11 | 64150370 |
| rs56167332 | A | C | 0.3375 | 0.1559 | 0.0105 | 7.17E-50 | A | C | 0.3509 | 0.0003 | 0.0072 | 0.9711 | 5 | 158827769 |
| rs6058869 | T | C | 0.3991 | 0.0557 | 0.0100 | 2.63E-08 | T | C | 0.4058 | -0.0089 | 0.0068 | 0.1928 | 20 | 31348750 |
| rs6074022 | T | C | 0.7497 | -0.0743 | 0.0114 | 8.32E-11 | T | C | 0.7364 | -0.0135 | 0.0077 | 0.0784 | 20 | 44740196 |
| rs6111031 | T | C | 0.1591 | -0.2641 | 0.0148 | 1.23E-71 | T | C | 0.1289 | 0.0087 | 0.0100 | 0.3825 | 20 | 1682037 |
| rs62434177 | A | G | 0.0324 | -0.1791 | 0.0314 | 1.14E-08 | A | G | 0.0346 | -0.0031 | 0.0204 | 0.8784 | 6 | 138087506 |
| rs6456426 | A | C | 0.4984 | -0.0643 | 0.0099 | 8.18E-11 | A | C | 0.4949 | -0.0136 | 0.0067 | 0.0424 | 6 | 21438889 |
| rs6466198 | T | A | 0.3860 | 0.0841 | 0.0102 | 2.18E-16 | T | A | 0.3920 | -0.0141 | 0.0070 | 0.0440 | 7 | 107480126 |
| rs648541 | G | A | 0.3409 | -0.0649 | 0.0107 | 1.22E-09 | G | A | 0.3206 | 0.0019 | 0.0071 | 0.7882 | 11 | 114429934 |
| rs6500315 | G | A | 0.7753 | 0.0766 | 0.0119 | 1.12E-10 | G | A | 0.7557 | 0.0114 | 0.0081 | 0.1585 | 16 | 50508101 |
| rs6561151 | A | G | 0.2235 | 0.1000 | 0.0119 | 3.53E-17 | A | G | 0.2048 | 0.0038 | 0.0081 | 0.6379 | 13 | 44484706 |
| rs6584281 | G | A | 0.5188 | -0.1646 | 0.0099 | 9.36E-62 | G | A | 0.5112 | 0.0024 | 0.0066 | 0.7184 | 10 | 101286480 |
| rs6588248 | G | T | 0.5297 | 0.0820 | 0.0099 | 1.38E-16 | G | T | 0.5250 | -0.0028 | 0.0066 | 0.6768 | 1 | 67652984 |
| rs6651252 | C | T | 0.1300 | -0.0908 | 0.0148 | 9.08E-10 | C | T | 0.1348 | -0.0027 | 0.0097 | 0.7832 | 8 | 129567181 |
| rs6708373 | G | A | 0.5277 | 0.1342 | 0.0099 | 1.43E-41 | G | A | 0.5193 | 0.0070 | 0.0066 | 0.2930 | 2 | 234172846 |
| rs6740462 | A | C | 0.7378 | 0.0800 | 0.0116 | 5.59E-12 | A | C | 0.7245 | 0.0027 | 0.0076 | 0.7198 | 2 | 65667272 |
| rs6745185 | G | T | 0.7389 | 0.0698 | 0.0115 | 1.37E-09 | G | T | 0.7458 | -0.0082 | 0.0079 | 0.2946 | 2 | 241586960 |
| rs67643815 | T | G | 0.5342 | -0.0629 | 0.0102 | 6.42E-10 | T | G | 0.5274 | 0.0004 | 0.0068 | 0.9571 | 18 | 67561508 |
| rs6933404 | C | T | 0.2108 | 0.0958 | 0.0123 | 5.84E-15 | C | T | 0.2104 | 0.0129 | 0.0082 | 0.1163 | 6 | 137959235 |
| rs7015630 | C | T | 0.2657 | -0.0628 | 0.0113 | 2.90E-08 | C | T | 0.2660 | -0.0027 | 0.0076 | 0.7230 | 8 | 90875918 |
| rs71593329 | G | T | 0.1998 | -0.0978 | 0.0127 | 1.19E-14 | G | T | 0.2077 | -0.0021 | 0.0083 | 0.8013 | 5 | 158620079 |
| rs7194886 | T | C | 0.4357 | -0.1260 | 0.0100 | 2.53E-36 | T | C | 0.4275 | -0.0040 | 0.0068 | 0.5513 | 16 | 50725193 |
| rs7240004 | G | A | 0.3795 | -0.0665 | 0.0103 | 1.01E-10 | G | A | 0.3794 | 0.0011 | 0.0069 | 0.8758 | 18 | 46395022 |
| rs7253253 | T | G | 0.9543 | -0.1344 | 0.0231 | 6.19E-09 | T | G | 0.9252 | 0.0312 | 0.0168 | 0.0631 | 19 | 10714058 |
| rs72924296 | G | A | 0.2696 | -0.0638 | 0.0113 | 1.44E-08 | G | A | 0.2729 | 0.0004 | 0.0076 | 0.9545 | 2 | 199543967 |
| rs744166 | G | A | 0.4204 | -0.1000 | 0.0102 | 1.14E-22 | G | A | 0.4382 | 0.0069 | 0.0067 | 0.2995 | 17 | 40514201 |
| rs7523442 | T | C | 0.5356 | 0.1245 | 0.0099 | 2.76E-36 | T | C | 0.5177 | 0.0135 | 0.0067 | 0.0447 | 1 | 20165971 |
| rs7547569 | C | T | 0.0668 | -0.6472 | 0.0233 | 1.65E-170 | C | T | 0.0653 | 0.0044 | 0.0139 | 0.7534 | 1 | 67731368 |
| rs7608910 | G | A | 0.3909 | 0.1264 | 0.0100 | 2.60E-36 | G | A | 0.3655 | 0.0087 | 0.0069 | 0.2064 | 2 | 61204856 |
| rs769177 | T | C | 0.0255 | 0.2609 | 0.0286 | 6.53E-20 | T | C | 0.0216 | -0.0004 | 0.0238 | 0.9857 | 6 | 31547611 |
| rs7711427 | C | A | 0.6130 | 0.1748 | 0.0102 | 4.63E-66 | C | A | 0.6037 | 0.0014 | 0.0101 | 0.8870 | 5 | 40414886 |
| rs7773324 | A | G | 0.6002 | 0.0618 | 0.0106 | 5.84E-09 | A | G | 0.6017 | 0.0028 | 0.0072 | 0.6980 | 6 | 382559 |
| rs7848647 | C | T | 0.6746 | 0.1324 | 0.0107 | 3.16E-35 | C | T | 0.6631 | 0.0152 | 0.0071 | 0.0327 | 9 | 117569046 |
| rs79980175 | C | A | 0.1360 | -0.0953 | 0.0148 | 1.30E-10 | C | A | 0.1305 | 0.0116 | 0.0098 | 0.2359 | 5 | 40521892 |
| rs8127691 | C | T | 0.6132 | -0.1143 | 0.0101 | 8.98E-30 | C | T | 0.6090 | 0.0139 | 0.0075 | 0.0640 | 21 | 45614860 |
| rs941823 | C | T | 0.7509 | 0.0830 | 0.0115 | 6.19E-13 | C | T | 0.7326 | 0.0005 | 0.0077 | 0.9530 | 13 | 41013977 |
| rs9457247 | T | C | 0.5398 | 0.0892 | 0.0102 | 2.48E-18 | T | C | 0.5273 | 0.0030 | 0.0066 | 0.6542 | 6 | 167392174 |
| rs9557207 | G | A | 0.2231 | -0.0878 | 0.0121 | 3.52E-13 | G | A | 0.2096 | 0.0045 | 0.0081 | 0.5837 | 13 | 100036418 |
| rs9889296 | A | G | 0.2723 | -0.1050 | 0.0113 | 1.35E-20 | A | G | 0.2938 | -0.0038 | 0.0073 | 0.6060 | 17 | 32570547 |

Abbreviation: EA, Effect Allele; OA, Other Allele; EAF, effect allele frequency; SE, standard error; SNP, single nucleotide polymorphism; Chr, Chromosome
