# Supplementary figures and images for: Does Chronic Intestinal Inflammation Promote Atrial Fibrillation: A Mendelian Randomization Study With Populations of European Ancestry
Source: Front Cardiovasc Med. 2021 May 10;8:641291. doi: 10.3389/fcvm.2021.641291 (PMC8141578; doi:10.3389/fcvm.2021.641291)

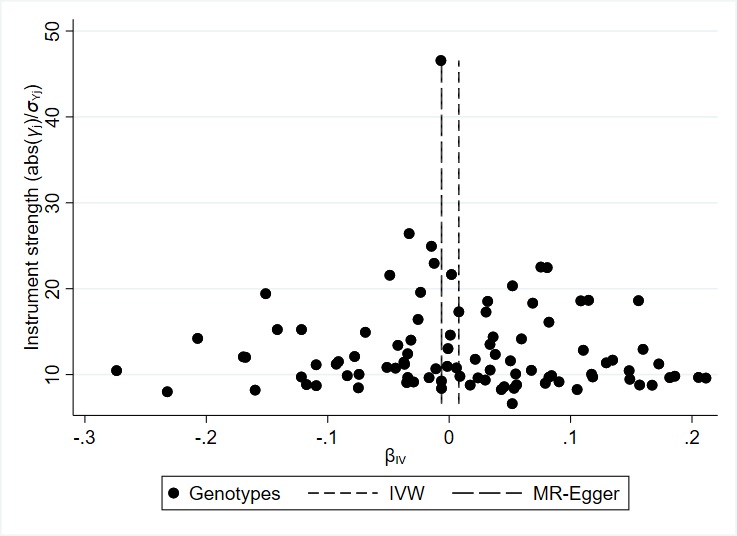

Supplement: Supplementary Figure 1 — Funnel plot of genetic associations between inflammatory bowel disease and causal estimates of atrial fibrillation. [file Image_1.jpg]

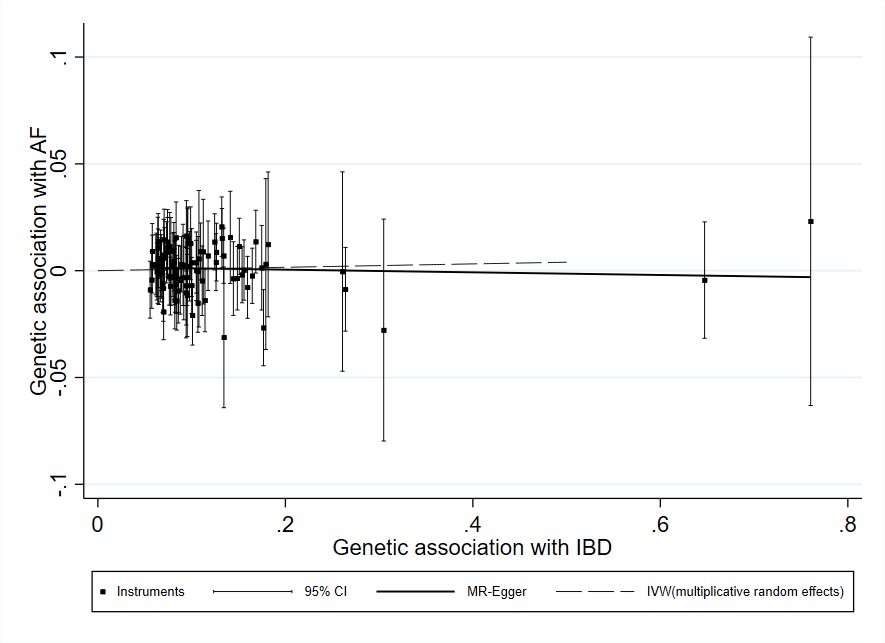

Supplement: Supplementary Figure 2 — Scatterplot of genetic associations between atrial fibrillation and inflammatory bowel disease. [file Image_2.jpg]

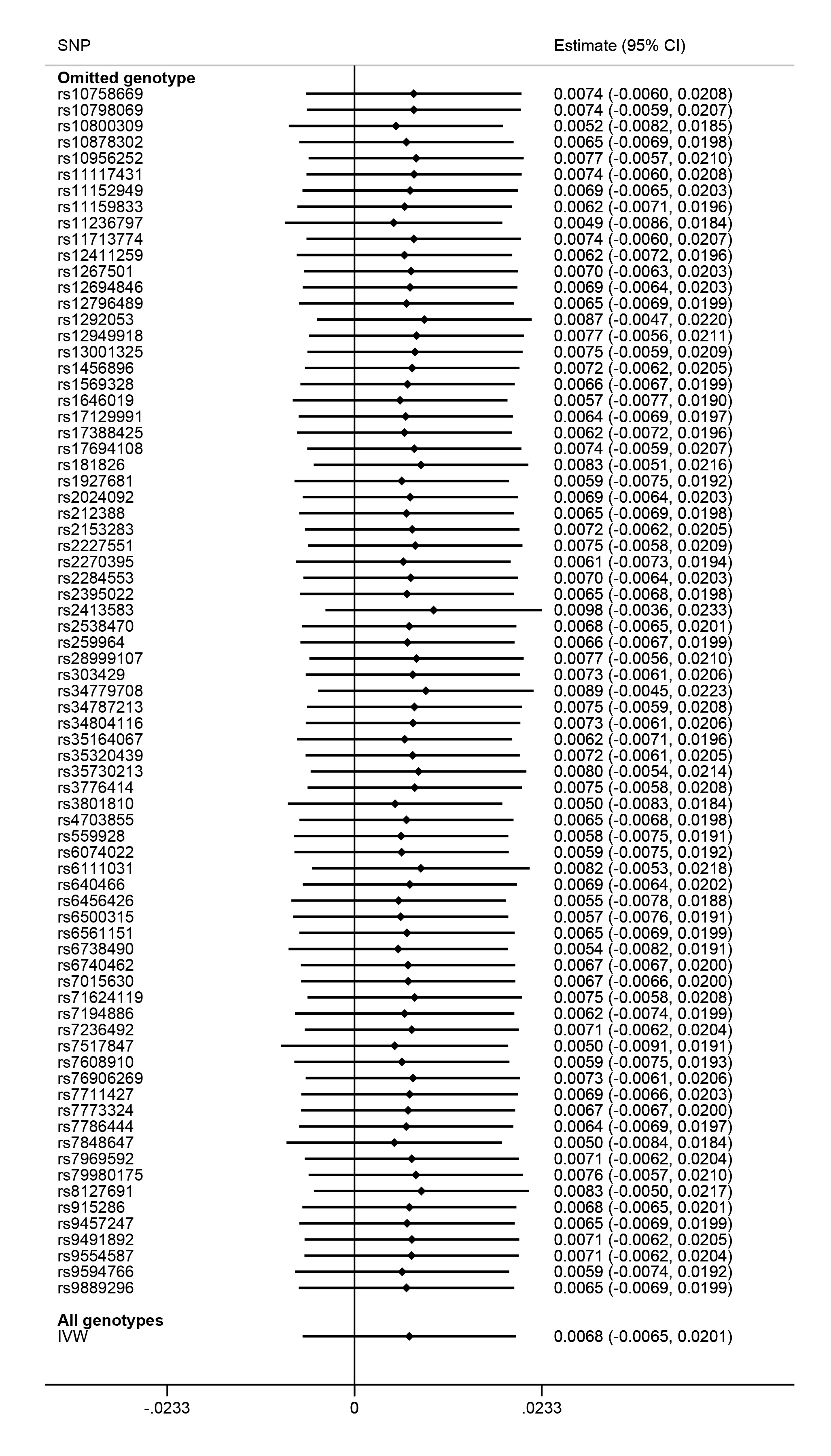

Supplement: Supplementary Figure 3 — Leave-one-out sensitivity analysis for causal effect of Crohn's disease on atrial fibrillation. The estimate is indicated by the odds ratio (OR). [file Image_3.jpg]

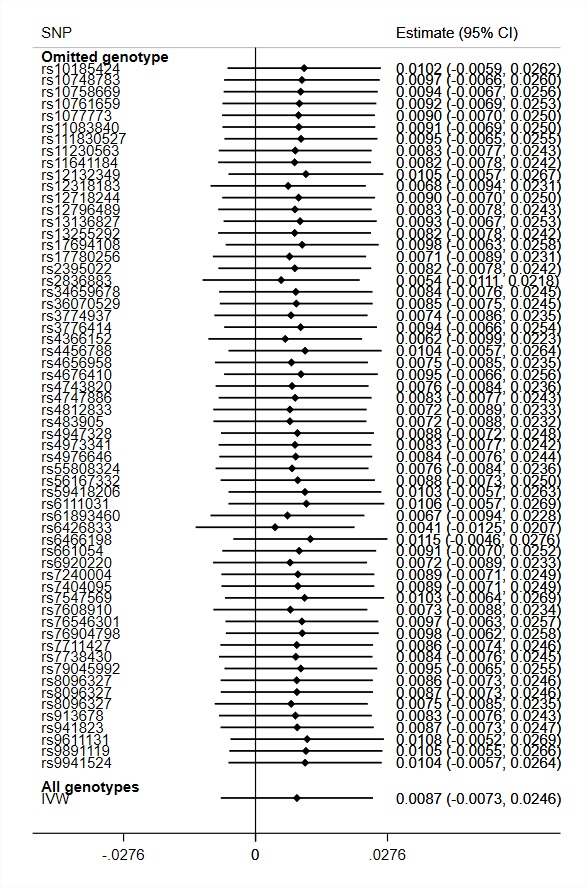

Supplement: Supplementary Figure 4 — Leave-one-out sensitivity analysis for causal effect of ulcerative colitis on atrial fibrillation. [file Image_4.jpg]

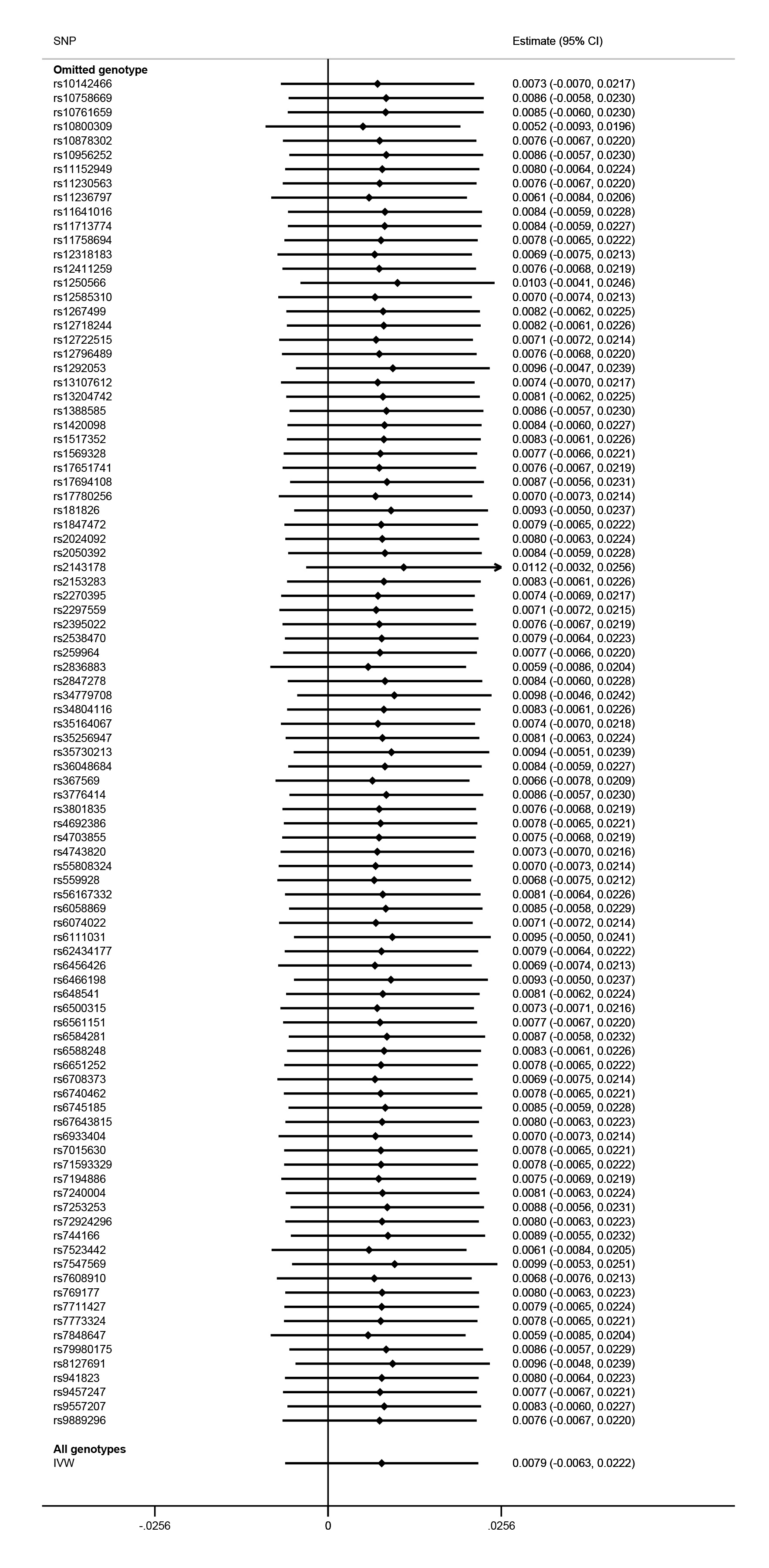

Supplement: Supplementary Figure 5 — Leave-one-out sensitivity analysis for causal effect of inflammatory bowel disease on atrial fibrillation. [file Image_5.jpg]
